# Using process evaluation for program improvement in dose, fidelity and reach: the ACT trial experience

**DOI:** 10.1186/1479-5868-6-79

**Published:** 2009-11-30

**Authors:** Dawn K Wilson, Sarah Griffin, Ruth P Saunders, Heather Kitzman-Ulrich, Duncan C Meyers, Leslie Mansard

**Affiliations:** 1 Department of Psychology, University of South Carolina, Columbia, SC, 29208, USA; 2 Department of Public Health Sciences, College of Heath, Education, and Human Development, Clemson University, Clemson, SC, 29634, USA; 3 Department of Health Promotion, Education, and Behavior, Arnold School of Public Health, University of South Carolina, Columbia, SC, 29208, USA

## Abstract

**Background:**

The purpose of this study was to demonstrate how formative program process evaluation was used to improve dose and fidelity of implementation, as well as reach of the intervention into the target population, in the "Active by Choice Today" (ACT) randomized school-based trial from years 1 to 3 of implementation.

**Methods:**

The intervention integrated constructs from Self-Determination Theory and Social Cognitive Theory to enhance intrinsic motivation and behavioral skills for increasing long-term physical activity (PA) behavior in underserved adolescents (low income, minorities). ACT formative process data were examined at the end of each year to provide timely, corrective feedback to keep the intervention "on track".

**Results:**

Between years 1 and 2 and years 2 and 3, three significant changes were made to attempt to increase dose and fidelity rates in the program delivery and participant attendance (reach). These changes included expanding the staff training, reformatting the intervention manual, and developing a tracking system for contacting parents of students who were not attending the after-school programs regularly. Process outcomes suggest that these efforts resulted in notable improvements in attendance, dose, and fidelity of intervention implementation from years 1 to 2 and 2 to 3 of the ACT trial.

**Conclusion:**

Process evaluation methods, particularly implementation monitoring, are useful tools to ensure fidelity in intervention trials and for identifying key best practices for intervention delivery.

## Introduction

Process evaluation can be used to explain why interventions succeed and fail, and whether there are characteristics or mechanisms involved in the program's implementation that potentially mediate or moderate outcomes. In large-scale trials the importance of monitoring program implementation has been highlighted [1-10] and there is strong evidence that level of implementation impacts study outcomes [4]. Implementation monitoring can be done in both a formative and a summative manner. Formative evaluations can be defined as utilizing data to provide on-going monitoring and quality assessment to maximize the performance of a program [11-14]. Summative evaluations analyze data at the conclusion of an initiative to provide a conclusive rating of the extent to which intended outcomes were achieved and the program was implemented as intended [11,13,14]. Another summative purpose of process evaluation is to include level of implementation data in the outcome analysis [15,16].

Evaluations of implementation are especially important given that few studies have achieved full implementation in real-world settings[16]. This is also true of health promotion efforts, as researchers have noted the great variability in program implementation and policy adoption in community and school settings [1,17]. Thus, one purpose of implementation monitoring is to ensure that the originally designed intervention is, in fact, being implemented, as well as being implemented in a manner that is consistent with the program theory and plan. In effect, if a complex intervention carried out in a field setting is not carefully monitored and adjusted to stay "on track" with the original plan, many different interventions may be implemented. Thus, midcourse changes are designed to increase fidelity, dose, and reach to enable researchers to evaluate the intervention as originally planned. Despite the importance of such evaluations, outcome analyses are frequently conducted without an assessment of program implementation [18]. This is often referred to as the "black box" approach to evaluation, which refers to examining the outcomes of a program without examining its internal operation. Lack of this knowledge can lead to "a Type III error," which refers to the conclusion that a seemingly ineffective program was, in actuality, not implemented as intended [19,20].

Process evaluation data used for formative purposes during a developed intervention, as described in this study, should be distinguished from process evaluation used for formative evaluation during the developmental phases of an intervention [21-23]. In an example of the latter, Wilson and colleagues [24] conducted a formative evaluation of a motivational PA intervention (Active by Choice Today; ACT). The conceptual framework for the ACT intervention targeted the social environment, cognitive mediators, and motivational orientation related to PA in underserved adolescents. The 8-week program sought to increase moderate-to-vigorous PA (MVPA) for participant youth, and formative evaluation was collected through daily forms and observational data completed by an independent objective observer. ACT process evaluation focused on identifying factors in the social environment and curriculum that worked well and/or were in need of improvement. Most effort was spent ensuring that the theoretical underpinnings of the program were maximized and promoting efficiency by modifying logistical flaws. The process evaluation was used to inform necessary changes to the staff training. Specifically, process data indicated that it would be more beneficial to encourage staff to praise students in subtle ways or in a setting where other students would not be aware of it (due to reduction in positive student-to-student reactions when publicly praised for their behavior by staff). The investigators also learned that training should focus on instructional methods which foster a balance between discipline and nurturing as well as ways to subtly dismantle cliques.

A growing literature has included process evaluation as a key element in evaluating success of implementation in large-scale PA trials. The Pathways initiative - a large-scale, multi-site, 3-year study testing a school-based intervention, used process evaluation methods in evaluating implementation of an intervention to lower percent body fat in American Indian children [25]. Pathways applied a multilevel strategy involving individual behavior change and environmental modifications to support changes in individual behavior. The environmental component included a food service intervention to enhance food staff skills in preparing and serving lower-fat meals. For this component, implementation was measured by various behavioral guidelines (e.g., use of low-fat vendor entrees, offer choice of fruits and vegetables). In the first year, none of the 12 goals were achieved; in the second year 6 of the 13 goals were met (a new goal had been added); in the third year 9 of the 13 goals were met. These improvements were due to performance feedback provided by the evaluators at the end of each semester, an example of effective use of formative process data.

Other large trials have reported summative process evaluations which have implications for using process evaluation data for formative purposes. For example, in one investigation of the SPARK program (Sport, Play, and Active Recreation for Kids), a multi-component elementary school program which sought to promote PA in elementary children, process evaluation data was obtained to determine success of implementation[26]. The SPARK curriculum focused on physical education (PE) and self-management (SM), and children participated in either an intervention implemented by PE specialists, an intervention implemented by classroom teachers, or a control (usual PE classes). Through direct observation of weekly classroom lessons it was determined that teachers and PE specialists conducted 63% and 67% of the components of the SM curriculum, respectively. The small variance in intervention delivery coupled with the relatively low implementation percentages suggests the possibility of consistent contextual implementation barriers that perhaps could have been addressed with timely, formative process evaluation data.

In "Switch-Play," Salmon and colleagues [27] sought to reduce the time spent by primary school children in sedentary behaviors and to increase their skills in, enjoyment of, and participation in PA outside of school. The process evaluation indicated an average attendance of 88% among children in the intervention conditions. Classroom activities were completed 92% of the time; however, outside-of-class PA activities and self-monitoring sheets were completed 57% and 62% of the time, respectively. These data indicate opportunities for improving fidelity to essential program elements, especially for outside of class PA.

The purpose of the present study was to demonstrate how program process evaluation was used in a formative manner [11] to improve fidelity and dose (completeness) of implementation as well as reach into the target population in the ACT randomized school-based trial from year to year of implementation. The ACT trial [28], is a group-randomized cohort design with three intervention and three comparison schools per year over the course of four years (N = 24 schools, n = 60 6^th ^graders per school). The formative data from each year were used to provide corrective feedback to keep the intervention "on track", and was part of a comprehensive approach to process evaluation for monitoring and assessing program implementation in ACT [28].

## Methods

### Participants

A total of 24 middle schools (range of 41-71 students per school; N = 1,422 total students) in South Carolina were recruited to participate in one of the two after-school programs (ACT intervention or a general health program that served as a comparison program) over the 4 years (6 schools per year) of the trial implementation. To be eligible, adolescents were required to 1) currently be enrolled in the 6^th ^grade, 2) have parental consent to participate, 3) agree to study participation and random assignment, and 4) be available for a 6-month follow-up. Adolescents were excluded from participation if they 1) had a medical condition that would interfere with the prescribed PA intervention plan, 2) were developmentally delayed such that the intervention materials would not be cognitively appropriate or, 3) were currently in treatment for a psychiatric disorder.

### Study Design

The ACT trial is a group-randomized cohort design with three intervention and three comparison schools per cohort (year). The schools were paired prior to recruitment and randomization to condition to avoid possible bias or confounding by socio-demographic differences. The criteria on which the schools were paired included: 1) school size, 2) proportion of minority versus non-minority ethnicity, 3) proportion of students enrolled in free and reduced lunch program and 4) urban or rural community setting. Baseline psychosocial, PA, and anthropometric measures were obtained prior to randomizing schools in each pair. The measurement team and intervention team maintained separate entities to blind the measurement staff to group conditions. Data was collected by trained measurement staff for each pair of schools on the same days over a period of two weeks in a lagged timeline (pair 1, pair 2, pair 3, respectively). This paper reports on years 1, 2, and 3 of the trial.

### Recruitment

Two phases of recruitment were implemented yearly during the ACT trial. The first phase involved attending parent orientations at school events to provide program information and obtain informed consent. Following the orientation a second phase of recruitment took place during the school day. Pep rallies and homeroom visits were two methods of recruitment implemented during the second phase to increase enrollment and excitement about the programs (PA and general health education). Randomization of schools to programs (PA intervention vs. general health education) occurred after recruitment and baseline assessments were completed. The recruitment target was 60 students from each school.

### ACT Intervention

The intervention integrated constructs from Self-Determination Theory (SDT) [29,30] and Social Cognitive Theory (SCT) [31] to enhance intrinsic motivation and behavioral skills for increasing long-term PA behavior specifically in underserved adolescents. A formative evaluation of the theoretical elements was developed during year 1 of the ACT trial [24,28]. In the present study elements from SCT and SDT were combined to develop an intervention that promoted behavioral skills for PA outside the program and a social environmental approach during the after-school program for enhancing autonomy (choice), fun, belongingness (engagement), and competence (challenges emphasizing non-competitive play) for PA [28]. An interview methodology known as strategic self-presentation was used to integrate SDT and SCT by linking motivational elements from the program to applying behavioral skills for being physical active outside of program time.

Investigators and staff defined the "essential elements" for the ACT intervention guided by constructs from the ACT theoretical frameworks (SDT and SCT) [28]. The essential elements informed the development of the program (e.g., ACT program content, methods, and activities), guided staff training, and defined dose (completeness) and fidelity for ACT intervention implementation. The program components and essential elements were also phrased into a list of concise terms that was used in training and to convey the philosophy and approach of the program in a "user-friendly" manner. Table 1 presents the theoretically-based elements of the ACT intervention and the ACT essential elements, including the "user-friendly" terms. Collectively, the essential elements of the intervention were designed to increase perceived competence, intrinsic motivation, commitment, and positive self-concept.

**Table 1:** ACT Theories, Theoretical Constructs, and Essential Elements.

Theory	Theoretical construct and definition	ACT Essential Element	Key Word
SDT	Autonomy supportive environment-contributes to feelings of agency or being "in control" ("internal locus of causation")	Participants have choices	Input and Choice
		Participants provide meaningful input, have influence on what happens in program	
		Participants know what is expected of them	Successful and confident (in program)
SDT	Competence supportive environment-contributes to being able to effectively interact with ones environment and get wanted effects and outcomes	Participants feel capable and able to participate successfully	
		Participants are engaged and involved in program	Engaged and Interact
SDT	Relatedness supportive environment-contributes to feelings of connectedness and being accepted by significant others	Participants feel that they belong and are part of the group	Belonging
		Participants feel like they are a valued member of group	
		Participants get along with each other, show respect for each other (positive interactions)	Respect
SDT	Intrinsic motivation for physical activity	Participants enjoy being in the program and being physically active	Fun & Enjoyment
		Participants are physically active during the PA component of the program	Being physically active
SCT	Self efficacy (person factor)-confidence in ones ability to successfully engage in a behavior	Participants feel confident that they can be physically active in the program, and at home	Successful onfident (at home)
SCT	Behavioral skills (behavioral factor)-skills or capability to self-regulate behavior (self-monitoring, group goal setting, support seeking)	Participants have specific behavioral skills that enable them to be physically active at home	Life skills
SCT	Social support (environmental factor)-instrumental and/or emotional support from peers and/or family members to engage in a specific activity	Participants have the social support needed to be physically active at home	Support
SDT & SCT	Self concept/motivation- Participants have a self-concept that includes being physically active	Students participate in strategic self presentation	Self-motivated

Note: SDT = Self-Determination Theory; SCT = Social Cognitive Theory; ACT = Active by Choice Today

The ACT intervention was implemented on Mondays, Tuesdays, and Thursdays for two hours after school. The ACT intervention was supervised by a team leader who had expertise in implementing physical activities in youth. The team leaders provided the structure for the ACT intervention components including the PA component. Four additional trained staff provided oversight and assisted with facilitating the program components. The program had three main components: snack/homework (30 minutes), a PA component that included activities which the students selected each week of MVPA (60 minutes), and a SCT and motivational component (group time/behavioral skills) during which intervention staff taught participants behavioral skills and motivational strategies to increase their PA at home and with friends (30 minutes).

The General Health Education Program (comparison program) focused on nutrition, stress management, drug prevention, and school drop-out prevention. The program was held on the same days and times as the ACT intervention program. The health education modules were taught in an interactive format and students typically rotated from one station to then next every twenty minutes [32].

### ACT Intervention Training

ACT intervention staff and volunteers were trained prior to the beginning of intervention each school year and received one booster training session midway through the intervention period. Training content included: an overview of the ACT trial purpose, an introduction of the behavioral theories and models guiding the ACT intervention, a detailed review of the ACT intervention manual, staff expectations regarding implementing the intervention and record keeping, team building, interacting with students, first aid, and administrative responsibilities and procedures. Training sessions were didactic and interactive. The interactive components provided opportunities for the staff to practice intervention strategies and for training leaders to identify and correct any problem areas for the staff during the training.

### ACT Process Evaluation Methods

ACT process evaluation methods were guided by the essential elements framework that defined dose and fidelity or "complete and acceptable delivery" of the ACT intervention [24,33,34]. The essential elements described in Table 1 guided the development of items for the process evaluation observation form; that is, the key concepts reflected in Table 1 were reflected throughout the components of the ACT intervention *as implemented during the after-school program*. The evaluation questions, presented below, guided the selection of methods and tools: 1) Fidelity (for PA and behavioral skills components)- To what extent was the social environment autonomy supportive?, 2) Dose delivered (completeness for all components)-To what extent were all planned components of the program provided to program participants? and 3) Reach-What percentage of the possible target group attends each week of the program?

Process evaluation data were collected by a trained, independent process evaluator using systematic observation of after-school program activities. Through observation and use of a quantitative checklist and ratings scales, the process evaluator assessed the extent to which the ACT after-school social environment achieved the essential elements upon which the program was designed. To assess dose and fidelity, the process evaluator observed the two-hour program for each day of the program for two weeks (3 program days for two weeks) at three points in time, early (weeks 1 and 2), midpoint (weeks 8 and 9) and near the end (weeks 15 and 16) of the 17-week program. It was possible to observe each program in the same phase of implementation because program implementation was staggered by 2 weeks across the three intervention sites.

An overview of the fidelity and dose process tools is provided in Tables 2 and 3, respectively. As shown in Table 2, observational data capturing fidelity was scored on a 4 point scale with 1 representing lowest fidelity and 4 representing highest level of fidelity. Fidelity measures for the PA and behavioral skills component of the program included measures for clarity of rules and expectations, choice, optimal challenge, relatedness and belonging. Mean scores were used to summarize the results. An overall mean was calculated to reflect overall fidelity for each school, based on six weeks of program observation, as noted above.

**Table 2:** Intervention Process Evaluation Form for Assessing Fidelity for the PA and Behavioral Skills/Group Time components

Program Component	Essential element categor *y and Sample item*	# of Items	Format
Physical Activity	1) Clarity of Rules/Expectations *Explain rules and daily activities to students*	1) 3	Likert Scale: 1-4
	2) Choices *Students get to vote on physical activity games*	2) 3	*(1 = none, 2 = some, 3 = most, 4 = all)*
	3) Optimal Challenges *Leaders encourage participation, fairness and de-emphasizes competition*	3) 8	
	4) Relatedness/Belonging *Leaders create a positive, interactive environment*	4) 6	
	5) Physical Activity *Students are participating in moderate to vigorous cooperative PA activities*	5) 5	
Behavioral Skills/SSP/Group Time	1) Clarity of Rules/Expectations *Explains rules to students*	1) 3	Likert Scale: 1-4
	2) Optimal Challenges *Leaders encourage participation and individual making progress (based on goals)*	2) 7	*(1 = none, 2 = some, 3 = most, 4 = all)*
	3) Relatedness/Belonging *Leaders create a positive, interactive environment*	3) 7	

Note: SSP = Strategic Self-Presentation

Many implementation fidelity ratings reported in the literature pertain to implementation or a curriculum or set of program activities and a rating ranging from "poor to excellent" has typically been optimal [11,13]. In the ACT trial, however, a different approach to conceptualizing and measuring fidelity was used given the goal of the intervention was to create a positive social environment in the program that was characterized by adult staff behavior. This approach was based on SDT [29,30] and because adult behaviors shape the program environment for the child, we selected a rating ranging from "all to none" to assess appropriate staff behavior.

As shown in Table 3, the dose assessment used yes/no response options; frequencies and percentages of "yes" responses were used to summarize the results. An overall percentage score was calculated to reflect overall dose delivered for each school. In addition, daily attendance was recorded by the team leader at each school during program days. These records were faxed weekly to the project director.

**Table 3:** Description of Intervention Process Evaluation Form for Assessing Dose (or completeness of delivery) for the PA and Behavioral Skills/Group Time components

Program Component	Essential elements	# of Items	Format
Dose for Snack/Welcome	Greeted arriving students Snack served Ground rules displayed Staff arrive on time Staff perform assigned duties Adult leader gave overview of week (Monday only) Adult leader gave daily overview	7	Yes/No
			
Dose of Physical Activity	Overview and introduction to entire activity session PA choices listed everyday Warm-up at beginning of PA session Activity introduced Cool-down	5	Yes/No
			
Dose of Behavioral Skills/SSP/Group Time	Overview of session or activity Topic/Skill explained Demonstration of skill Student involvement (brainstorm, role play, etc) Summary/closure	5	Yes/No

After the intervention was completed each year, the process evaluation data were examined to determine areas of strengths and weaknesses and to make adjustments to keep the program "on track" for the next year (cohort). Based on process evaluation data in year 1, changes were made in the subsequent program years to ensure complete and acceptable program delivery and to maximize reach into the target population.

## Results

### Demographics

Demographic data for participants in years 1, 2, and 3 are provided in Table 4. Students ranged from 10 to 14 years of age with an average age of 11.39 years. Just over half (55%) of the participants were female, 73% were African American and 76% qualified for the free or reduced lunch program through the schools.

**Table 4:** Student Demographics by Year and Intervention vs. Control Schools

Demographic	Control Schools			Intervention Schools			All Schools
**Year**	**1**	**2**	**3**	**1**	**2**	**3**	**All**

Age							
Mean	11.41	11.44	11.31	11.41	11.36	11.33	11.37
Range across schools	10-13	10-14	10-13	11-13	10-13	11-13	10-14
Gender							
% Female	55.13	55.83	53.85	51.85	57.49	61.45	55.93
Range across schools	52.5-58.5	48.3-60.8	52.4-55.4	50-53.7	55.4-60.0	59.3-65.0	48.3-65.0
Race							
% African American	74.36	71.17	71.43	69.20	80.00	75.98	73.69
Range across schools	63.6-86.4	56.7-84.3	46.0-90.8	50.0-90.7	73.8-88.1	66.7-88.1	46.0-90.8
Free or Reduced Lunch							
% F/R	70.10	75.50	69.30	85.8	81.30	79.00	76.83
Range across schools	62.3-81.4	73.1-80.4	64.9-76.1	64.8-83.9	69.2-98.2	58.1-90.5	58.1-98.2

### Recruitment and Attendance

All but one school met the recruitment minimum goal of 60 participants. As shown in Table 5, intervention school attendance in year 1 shows the average attendance per school ranged from 40% to 51% (schools are denoted by number from the order in which they were worked with during years 1-3 of the trial). Intervention schools had slightly higher attendance than comparison schools (e.g. general health program). Overall attendance rates slightly improved in years 2 and 3 for intervention schools, however attendance remained fairly constant for comparison schools.

**Table 5:** Average Attendance Summary by Year for Intervention and Control Schools

School	PA Intervention % attendance			Control % attendance			Total %	
							PA	C
Year 1	40	48	51	46	49	47	46	47
Year 2	59	78	55	42	39	44	64	42
Year 3	67	55	48	55	41	49	57	48

### Tracking System Changes

In response to attendance challenges in year 1, a tracking system was developed to more easily contact parents whose children had poor attendance at ACT. Detailed protocols were developed for ACT and general health intervention participants. The protocols included detailed phone scripts and follow-up actions for various scenarios (e.g. wrong phone number, no answering machine, leaving a phone message). The information was then included in a tracking database that included codes for the various scenarios. Staff attempted to collect updated contact information if it was not readily available from the school or provided by the participant.

### Intervention Dose and Fidelity

The goal of implementation monitoring in ACT was to reach implementation criteria/goals and to ensure complete and acceptable delivery of ACT by criteria that were determined prior to program implementation. Dose delivered (completeness of program delivery) and fidelity were captured through 49 observations in year 1, 47 observations in year 2, and 48 observations in year 3. As shown in Table 6, in year 1 the completeness rate ranged from 60% to 75% for snack, 32% to 80% for PA, and 48% to 75% for behavioral skills components. The goal was 75% or higher overall, which two schools did not attain. Two of the three schools were rated low in "snacks". The snack is critical to "setting the stage" for the day as it "welcomes" the participants and lets the participants know what will happen each day. Therefore it was important for ensuring that the core content was implemented each day. The areas most commonly omitted during the afterschool interventions involved posting ground rules and providing overviews. For PA, the most commonly omitted elements were explanations of activities, demonstration of skills, and summaries. These are most related to the intervention element of "clarity of rules and expectations". Several strategies to improve dose, described below, were implemented after year one. This seemed to result in improvements for years two and three, as reflected in Table 6. The remaining areas of weakness were mainly in the snack/welcome component at the beginning of each program day (problems: greeting students in all three schools, posting ground rules in two schools, providing an overview of the week in 1 school for year 2, and providing the summary or closure element in the group time/behavioral skills components.

**Table 6:** Percentage of Dose Delivered for ACT Intervention Components Cohorts 1, 2 and 3 (Goal 75% or higher)

	Cohort 1 Schools			Cohort 2 Schools			Cohort 3 Schools		
School number	1	2	3	4	5	6	7	8	9
Snack (7 elements)	60	75	65	87	91	77	91	94	93
Physical Activity (4 elements)	80	73	32	100	97	97	98	100	99
Beh. Skills (4 elements)	70	75	48	92	90	85	100	100	100

Average	70	74	48	93	93	86	96	98	97

Note: Schools are denoted by number from 1-9 representing the order in which we worked with the schools during years 1-3 of the trial.

### Staff Manual Changes

There were both curricula as well as visual and organizational changes made to the manuals. The curricula changes included not repeating any weeks during the program. In addition, some activities were taken out that weren't feasible. For example, a camera activity was taken out because it was not feasible to give each child in each school a camera to complete the activity. Visual and organizational changes were also made to the manual. Each daily sheet was changed to include a "to do" list. A "what's the point?" box was added near the top to reinforce top priorities for each daily activity, and which ACT essential element was being covered that day. Fun and interesting visuals were also added to make the daily sheets more appealing to ACT staff; who were primarily school teachers and staff. Finally, important points conveying the main emphasis of ACT (i.e. fun, belongingness) were bolded and functional definitions were added where appropriate.

As shown in Table 7, fidelity data from year 1 indicated some problems. Elements that needed improvement included "clarity of rules and expectations" for PA session and group time/behavioral skills, as well as "optimal challenges" for group time/behavioral skills. Fidelity improved from years 1 to 2, especially choices in the PA component, and clarity of rules and expectations and optimal challenges in group time/behavior skills component. Areas that remained high (e.g., were implemented to a high degree) from years 1 to 2 were optimal challenges, relatedness and belonging in the PA component, and relatedness and belonging in group time/behavioral skills component. Areas that continued to need improvement for all schools are clarity of rules and expectations for both PA and group time/behavior skills components. Areas of weakness from years 1 and 2, clarity of rules and expectations for both PA and group time/behavior skills components and PA during the PA component, were improved in year 3.

**Table 7:** Summary of Fidelity Scores for ACT Intervention Components-Cohorts 1, 2 & 3 (Goal 3 or higher; Scale 1-4)

	Cohort 1 Schools			Cohort 2 Schools			Cohort 3 Schools		
School number	1	2	3	4	5	6	7	8	9
Physical Activity (10 items)	3.0	3.2	2.7	3.5	3.3	3.0	3.7	3.7	3.7
Beh Skills (6 items)	2.5	2.9	2.6	3.4	3.2	3.2	3.7	3.6	3.9

Average	2.7	3.0	2.6	3.4	3.1	3.2	3.7	3.7	3.8

Note: Schools are denoted by number from 1-9 representing the order in which we worked with the schools during years 1-3 of the trial.

### Staff Training Changes

Significant changes were made in staff training to attempt to improve program dose and fidelity. A core-training with all the schools team leaders was developed and implemented prior to any of the programs start dates. In this training, team leaders spent 20 hours being exposed to all the essential elements of ACT. They participated in hands-on activities that helped them become more familiar with the basic elements of the program. After the core training, team leaders then helped facilitate their school's staff training. The team leaders took on a more active and leadership role in these 12-hour school trainings. Mid-year, a booster training session was held and feedback was given to each team staff member by the ACT project director. Constructive feedback was given based on internal evaluations that had been conducted by the project director. Finally, the external evaluator's criteria sheet was shared with staff members so that they would become familiar with exactly how the essential elements of the program were translated into specific staff tasks and responsibilities.

## Discussion

Overall, this study suggests that the formative evaluation contributed to improving the intervention dose, fidelity, and program attendance. The intervention itself was not changed; rather, the changes made enabled ACT staff to do a better job of delivering the planned intervention. Many of the changes were related to staff training and monitoring methodology. Specifically, changes in the staff training, the intervention manual, and tracking of students' participation were associated with reaching the goals for dose, fidelity, and reach when comparing years 1 through 3 of implementation. These findings have important implications for future research and suggest that formative process evaluation procedures can inform and enhance program implementation in on-going trials.

Using process evaluation data in a formative manner is frequently recommended; however, there are relatively few reports describing formative compared to summative uses of process evaluation. A commonly cited challenge, particularly in large trials, is the time frame required for data collection, management, synthesis, and reporting [14]. This includes the need to develop project infrastructure and procedures that enable project staff to get and use the information in a timely manner. Pre-implementation development of project "essential elements" that define dose and fidelity and a comprehensive process evaluation plan sets the stage and expectations for developing project infrastructure and process evaluation procedures to ensure program implementation and quality [20].

In a review conducted by Durlak and DuPre [4], it was demonstrated that inadequate implementation of a program can adversely affect program outcomes. This is particularly a concern for multi-component programs, given that an improperly implemented component will likely influence the implementation of another. Process data can help ensure that a program stays true to its underlying theory and plan. Theory not only informs proper and desired implementation, it conversely ties implementation to theory and maximizes the possibility of detecting desired outcomes. There is now evidence that links better PA outcomes to fidelity, and methods suggested in this paper may serve as a "best process practice" [34] that help practitioners identify aspects of PA interventions, [5,24,28] that may mediate or moderate positive outcomes.

## Competing interests

The authors declare that they have no competing interests.

## Authors' contributions

DKW (1^st ^author) is the Principal Investigator on the study and participated in the work of the whole content of this manuscript including conception, design, data acquisition, interpretation of data, drafting the manuscript, critical revision, and supervision. SG (2^nd ^author) is a Research Associate, Assistant Professor, who was involved in the conception and design, data acquisition, interpretation of data, drafting the manuscript, critical revision, and supervision. RS (3^rd ^author) is a Co-Investigator on the study and participated in the conception and design, data acquisition, analysis and interpretation of data, drafting the manuscript, critical revision, and supervision. HKU (4th author) is a Co-Investigator on the study and participated in data acquisition, drafting the manuscript, critical revision, and supervision. DM (5^th ^author) is a graduate student on the study who participated in data acquisition, and drafting the manuscript. LM (6^th ^author) is a Research Associate who participated in the acquisition of data, data analysis and interpretation, critical revision of the manuscript, and supervision.
